# Inflammatory myopathies: an update for neurologists

**DOI:** 10.1590/0004-282X-ANP-2022-S131

**Published:** 2022-08-12

**Authors:** André Macedo Serafim Silva, Eliene Dutra Campos, Edmar Zanoteli

**Affiliations:** 1 Universidade de São Paulo, Faculdade de Medicina, Departamento de Neurologia, São Paulo SP, Brazil.

**Keywords:** Myositis, Dermatomyositis, Polymyositis, Immune-Mediated Necrotizing Myopathy, Miosite, Dermatomiosite, Polimiosite, Miopatia Necrosante Imunomediada

## Abstract

Idiopathic inflammatory myopathies (IIM) are a heterogenous group of treatable myopathies. Patients present mainly to the rheumatologist and neurologists, complaining of acute or subacute onset of proximal weakness. Extramuscular manifestations may occur, including involvement of the lungs, skin, and joints. Classically, the diagnosis used to be made based on the creatine kinase level increase, abnormalities in electroneuromyography and presence of inflammatory infiltrates in the muscle biopsy. Recently, the importance of autoantibodies has increased, and now they may be identified in more than half of IIM patients. The continuous clinicoseropathological improvement in IIM knowledge has changed the way we see these patients and how we classify them. In the past, only polymyositis, dermatomyositis and inclusion body myopathy were described. Currently, immune-mediated necrotizing myopathy, overlap myositis and antisynthetase syndrome have been considered the most common forms of IIM in clinical practice, increasing the spectrum of classification. Patients previously considered to have polymyositis, in fact have these other forms of seropositive IIM. In this article, we reviewed the new concepts of classification, a practical way to make the diagnosis and how to plan the treatment of patients suffering from IIM.

## RESUMO

As miopatias inflamatórias idiopáticas (MII) são um grupo heterogêneo de miopatias tratáveis. Os pacientes procuram principalmente o reumatologista e o neurologista, queixando-se de início agudo ou subagudo de fraqueza proximal. Manifestações extramusculares podem ocorrer, incluindo envolvimento dos pulmões, pele e articulações. Classicamente, o diagnóstico era feito com base na elevação dos níveis de creatina quinase, anormalidades na eletroneuromiografia e presença de infiltrados inflamatórios na biópsia muscular. Recentemente, a importância dos autoanticorpos aumentou, e agora eles podem ser identificados em mais da metade dos pacientes com MII. A contínua melhora clínico-soropatológica no conhecimento do MII mudou a forma como vemos esses pacientes e como os classificamos. No passado, apenas polimiosite, dermatomiosite e miopatia por corpos de inclusão eram descritas. Atualmente, a miopatia necrosante imunomediada, a miosite de sobreposição e a síndrome antissintetase têm sido consideradas as formas mais comuns de MII na prática clínica, aumentando o espectro de classificação. Pacientes previamente considerados como portadores de polimiosite, na verdade, têm uma dessas outras formas de MII soropositivas. Neste artigo, revisamos os novos conceitos de classificação, uma forma prática de fazer o diagnóstico e como planejar o tratamento de pacientes que sofrem de MII.

## INTRODUCTION

Myopathies are a heterogeneous group of diseases that affect skeletal muscle tissue, classified into hereditary and acquired forms^1^. In clinical practice, acquired causes are the priority for identification, and must not be missed, because they are potentially treatable. This group includes toxic drug exposure myopathy, infectious conditions, endocrinopathies and, mainly, idiopathic inflammatory myopathies (IIM), also known as myositis, or systemic autoimmune myopathies^2^. 

IIM are characterized by muscle weakness as the main symptom, and muscle inflammation as the central physiopathology^3^. Extramuscular manifestations may occur, including involvement of the lungs, skin, and joints^4^. Classically, some subtypes of IIM are described: dermatomyositis, polymyositis, immune-mediated necrotizing myopathy (IMNM), inclusion-body myositis (IBM), and overlap myositis, wherein some authors include antisynthetase syndrome, whereas other authors categorize these as a separate group^3^ ^,^ ^5^. Here we reviewed the heterogenous group of IIM, regarding advances in its diagnosis, classification, and treatment options, in a practical approach, for general neurologists. 

## HOW TO PERFORM THE DIAGNOSIS

Patients with IIM will present with acute or subacute onset of proximal weakness, usually over several weeks or a few months, with little or no atrophy, normal tendon reflexes (unless the patient has a severe muscle weakness), and normal sensory tests. The patients suffer from impaired walking, getting up from the chair and climbing stairs as well as lifting their arms. Dysphagia and neck weakness may occur in around one-third of patients, then a dropping head may be a clinical presentation^3^ ^,^ ^6^ ^,^ ^7^. Although frequently considered as a symptom of IIM, muscle pain or tenderness are not common, but may be present in some forms of IIM with perifascial involvement, such as dermatomyositis^8^. Isolated muscle pain with normal physical examination is not evidence of IIM. In patients with IBM, a progressive course disease, atrophy in the quadriceps and finger flexors, with hand weakness, are the clinical hallmarks, which is different from other IIM^9^. 

**Figure 1.**
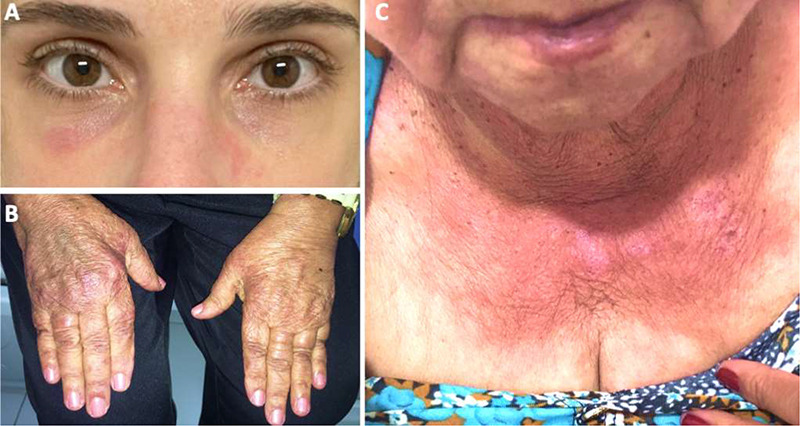
Skin manifestations of dermatomyositis. A) Reddish purple rash on the face, predominantly in periorbital region. B) Violaceous rash on the dorsal aspect of interphalangeal joints (Gottron’s sign). C) Anterior chest rash (V-sign).

In the clinical history, signs and symptoms of other organs and systems must be examined, especially skin lesions, such as periorbital blue-purple rash, reddish rash on the face, anterior chest (V-sign), and shoulders (shawl sign), and a violaceous eruption on the knuckles (Gottron’s papules), which are characteristics of dermatomyositis (Figure 1)^3^. Pulmonary complications, due to interstitial lung disease, may occur in 10 to 40% of patients, as well as fever, arthralgia, scleroderma, and Raynaud’s phenomenon, mainly in overlap and antisynthetase syndrome, defined as the presence of myopathy, fever, interstitial lung disease, Raynaud’s phenomenon, arthritis, and “mechanic’s hands”^10^. 

Creatine kinase (CK) is the main biomarker, usually over 10 times the upper limit of normality, but in some rare cases it is normal or just mildly elevated^7^. Aldolase is another muscle enzyme and may be the only elevated muscle enzyme in perimysial and perifascial inflammation^11^. Electroneuromyography (EMG) is not essential for the diagnosis, and is usually performed when there are doubts regarding differential diagnosis for myasthenic syndromes. In IIM, the EMG demonstrates motor potential of myopathic characteristics, with small motor unit potentials of short duration, and early recruitment^12^. Spontaneous activity, such as fibrillation and positive waves, is usually found, because there is active degeneration of muscle fibers, but this is not universal or specific^13^.

Recently, the importance and use of autoantibodies has increased, and they are identified in more than 50% of IIM patients^4^. Currently, commercial antibody panels for myositis are available. These autoantibodies may help in the diagnosis, classification of patients into more homogeneous groups, and prediction of additional clinical complications and treatment response^14^. Approximately 70% of dermatomyositis patients have one of the five dermatomyositis-specific autoantibodies^15^ ^,^ ^16^. Among them, anti-Mi2 is a well-known antibody related to classical skin lesion, high association with calcinosis and good response to immunosuppressant treatment^12^ ^,^ ^14^. Other dermatomyositis-specific autoantibodies are anti-TIF1-γ, anti-NXP-2, anti-MDA-5 and anti-SAE (Table 1). In addition, the discovery of autoantibodies related to antisynthetase syndrome, mainly anti-Jo-1, present in up to a quarter of IIM, allowed for the creation of a new subgroup of IIM, based on clinicoserological diagnosis^17^. Other autoantibodies in this syndrome have been described, such as anti-PL-7 and anti-PL-12 (Table 1)^18^ ^,^ ^19^. Anti-SRP and anti-HMGCR are present in most patients with IMNM, and have prognostic implications, because the first is more severe and resistant to immunosuppressors (Table 1)^7^. 

**Table 1.** Main myositis specific autoantibodies.

Dermatomyositis associated autoantibodies		
	Frequency †	Key characteristics
Anti-Mi2 (anticomplex nucleosome remodeling histone deacetylase)	~10%	Dermatomyositis, associated with typical skin lesions, less association with cancer
Anti-TIF1- γ (anti transcription intermediary factor 1 γ)	~20%	Dermatomyositis, severe cutaneous disease, strong cancer association
Anti-NXP-2 (antinuclear matrix protein 2)	~15%	Dermatomyositis, subcutaneous calcinosis, cancer association
Anti-MDA-5 (antimelanoma differentiation-associated gene 5)	~15%	Dermatomyositis, most patients are hypo myopathic or amyopathic, have atypical skin lesions, skin ulcers, arthritis, mechanic hands, interstitial pulmonary disease
Anti-SAE (antismall ubiquitin-like modifier-activating enzyme)	~5%	Amyopathic/hypo myopathic dermatomyositis
Antisynthetase syndrome associated autoantibodies		
	Frequency ‡	Key characteristics
Anti-Jo-1 (Anti-histidyl-transfer RNA synthetase)	~15%	Antisynthetase syndrome, progressive lung involvement and possible mild dermatomyositis skin rash
Anti-PL-7 (anti-analyl-transfer RNA synthetase)	~3%	Antisynthetase syndrome, more severe lung involvement
Anti-PL-12 (anti-threonyl-transfer RNA synthetase)	~3%	Antisynthetase syndrome, severe lung disease with mild muscle weakness
Others (Anti-EJ, Anti-OJ, Anti-KS, Anti-Zo, Anti-Ha)	~5%	Antisynthetase syndrome
Immune-mediated necrotizing myopathy associated autoantibodies		
	Frequency*	Key characteristics
Anti-HMGCR (Anti-3-hydroxy-3-methylglutaryl coenzyme A reductase)	~60%	Association with statin exposure (but in one-third there is no statin use), good response to immunosuppression, bur relapse after suspensions, also associated to chronic presentation
Anti-SRP (Anti-signal recognition particle)	~10%	Severity, dysphagia, refractory to immunosuppression, less association with statin exposure, possible cardiac involvement
Inclusion body myositis		
	Frequency#	Key characteristics
AnticN1A (Anticytosolic-5’nucleotidase 1A)	~30%	Associated with a more severe disease course and dysphagia. Other conditions such as Sjogren’s syndrome and systemic lupus erythematosus also present this antibody by ∼20-30%

†: estimated frequency among DM patients; ‡: estimated frequency among general myositis patients; *: estimated frequency among IMNM patients; #: estimated frequency among IBM patients.

**Figure 2.**
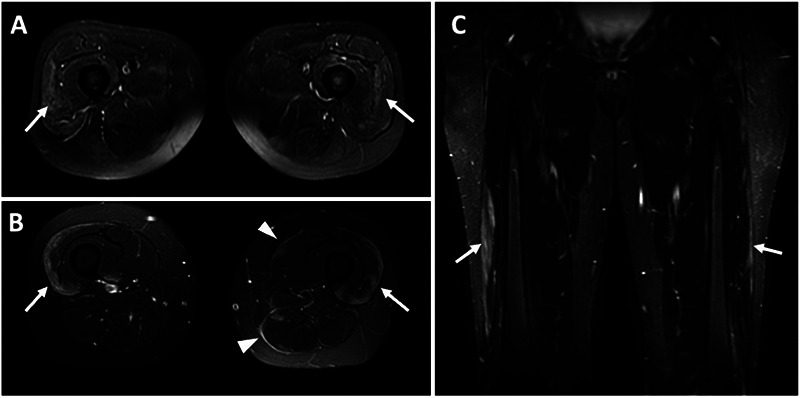
Magnetic resonance imaging in inflammatory myopathies. A) Axial STIR-weighted imaging of a patient with polymyositis with hyperintense areas of muscular edema, mainly in quadriceps (arrows). B) Axial STIR-weighted in a patient with dermatomyositis demonstrating a perifascial hyperintensity (arrows) and well-demarcated fascial hyperintense signal indicating fasciitis (arrowhead). C) Coronal STIR-weighted imaging with peripheral hyperintensity (arrows) in a patient with dermatomyositis.

Muscle imaging, mainly MRI, is now widely used as a biomarker for diagnosis and treatment follow-up. Magnetic resonance of the muscles demonstrates an increase in heterogeneous signals in the muscles in the T2- and STIR-weighted sequences, as well as an anomalous enhancement by gadolinium, indicating the presence of edema in the muscles (Figure 2A)^20^. Patients with dermatomyositis frequently present T2 / STIR hyperintensities in the perifascial region of individual muscles, a characteristic seen less often in other subtypes of IIM (Figure 2B-C)^21^. Although useful for differential diagnosis between hereditary and inflammatory myopathies, MRI was not included in the recent classification criteria for IIM. 

Muscle biopsy is still the gold standard for diagnosis, and is indicated in most patients, although it can be prevented for those with typical skin manifestation or known autoantibody syndromes (for instance, DM and ASS). Muscle biopsy continues to be essential for myositis-specific antibody-negative IIM and IBM diagnosis. Ideally, the analysis must include immunohistochemistry with antibodies against inflammatory cells, such as T and B lymphocytes (CD4, CD8 and CD20), macrophages (CD68), major histocompatibility complex (MHC) class I antigen and membrane attack complexes (MACs)^3^ ^,^ ^7^. Two techniques are used to obtain muscle samples: needle biopsy and open biopsy. Both are widely used, usually in the form of an outpatient procedure. We would prefer the open technique, because this has the advantage of obtaining a larger sample, under direct examination, allowing the collection of multiple fragments from different fascicles, increasing the diagnostic yield, especially in IIM, although it is more invasive, requiring a two to four cm incision^22^. 

**Figure 3.**
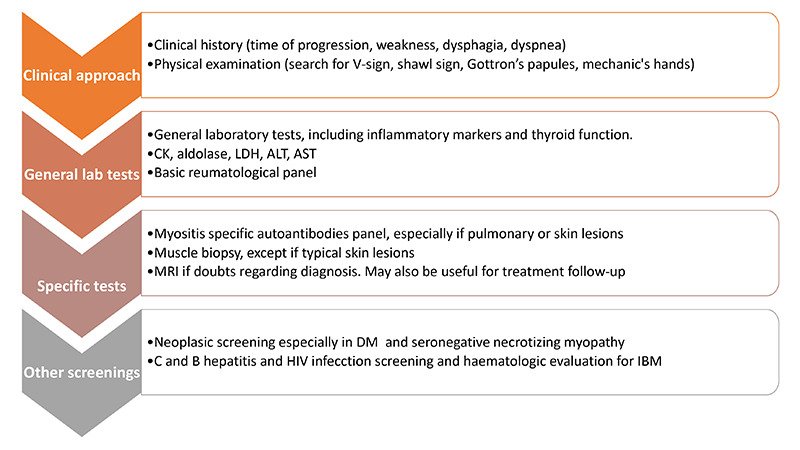
Flowchart for IIM diagnosis.

A proposed diagnostic approach for IIM is presented in Figure 3.

## HOW TO CLASSIFY THE PATIENTS

For many years, IIM had been classified into three subgroups, including polymyositis and dermatomyositis, based on the Bohan and Peter criteria^23^ ^,^ ^24^, and inclusion body myositis (IBM) using the Griggs criteria^25^. However, over the last decade, reviewed classifications, based on expert opinion, have been proposed^5^ ^,^ ^12^, with advances of imaging methods, better pathological descriptions, and discovery of myositis-specific autoantibodies (MSA)^14^. Newly identified autoantibodies are reclassifying some patients previously considered as dermatomyositis or polymyositis, now categorized as other forms of IIM^5^. 

IIM are currently classified into several subgroups: dermatomyositis (DM), polymyositis (PM), inclusion body myositis (IBM), immune-mediated necrotizing myopathy (IMNM), antisynthetase syndrome (ASS), and overlap myositis^5^ ^,^ ^7^ ^,^ ^12^ ^,^ ^14^ ^,^ ^26^. Some authors classify ASS into overlap myositis, whereas others categorize it as a distinct subform of myositis^7^ ^,^ ^12^ ^,^ ^27^. We prefer to present ASS as a distinct entity. An overview of the classification and the main characteristics of the IIM subgroups are shown here (Table 2). 

**Table 2.** Classification e key characteristics of the IIM subtypes.

DM	- Inflammatory myopathy accompanied by skin changes. - Some patients may have amyopathic or hypo myopathic presentations - There are five known autoantibodies associated: anti-Mi2, anti-TIF1-y, anti-NXP-2, anti-MDA-5 and anti-SAE - CD4 lymphocytes infiltrates, with a perivascular and interfascicular location and atrophy of the perifascicular fibers
PM	- No skin or pulmonary involvement - Good response to immunosuppressive treatment - No association with specific antibodies - Now considered an exclusion diagnosis - CD8 lymphocytes predominate, invading the endomysium and intact fibers
IMNM	- Associated with systemic conditions (cancer, statin, viral infections) - Presence of autoantibodies: anti-SRP and anti-HMGCR - In children, it may present as slowly progressive, and mimic muscular dystrophy - Abundant fibers in necrosis and macrophage predominance, which can be identified by labeling for CD68
IBM	- Slowly evolving weakness with distal atrophy in the hands and atrophy in the thighs - Individuals over 45 years - Association with anti-NT5C1A autoantibody - CD8 lymphocytes predominate, invading the endomysium and intact fibers and presence of marginated vacuoles
ASS	- Inflammatory myopathy, interstitial lung disease and joint involvement - Other findings: fever, "mechanic's hands" and Raynaud's phenomenon - All the patients have antibodies directed against aminoacyl-tRNA synthetases - The most common autoantibodies are anti-Jo-1, anti-PL-7, and anti-PL-12 - The muscle biopsy demonstrates T-cell and macrophage infiltrations and perifascicular atrophy and necrosis
OM	- Association of inflammatory myopathy with other connective tissue disorder - The most common antibodies are anti-PM /Scl and anti-U1-RNP - Perivascular inflammation, perifascicular necrosis and MHC-I increase

DM: dermatomyositis; PM: polymyositis’; IMNM: immune-mediated necrotizing myopathy; IBM: inclusion body myositis; ASS: antisynthetase syndrome; OM: overlap myositis.

### Dermatomyositis and amyopathic dermatomyositis

DM is a condition seen in adults and children, mainly females, characterized by cutaneous manifestations and proximal muscle weakness. Presentation includes generalized erythema, mainly in the upper region of the trunk (shawl or V sign), purplish lesion around the eyes (heliotrope), and violet eruptions on the finger joints (Gottron’s sign), all of which are photosensitive (Figure 1)^16^. Some patients develop an interstitial lung disease, Raynaud phenomenon and calcifications in muscles or skin, although the trend is to categorize them separately if an ASS-specific autoantibody is recognized^5^. 

There is an increase in CK levels at some stage of the disease in most patients. However, CK may be normal at the onset of the disease, after major muscle atrophies, or in a subgroup, representing around 5-10%, known as hypo myopathic or amyopathic, in which muscle weakness is mild or absent^7^. In these patients, anti-MDA5 or anti-SAE autoantibodies are more likely to be present^5^.

**Figure 4.**
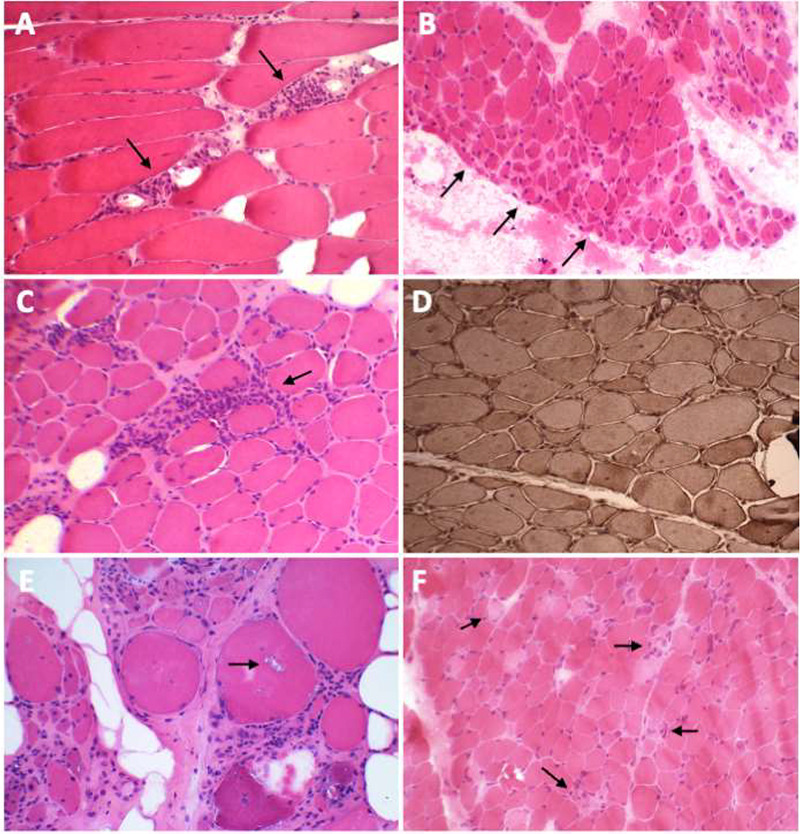
Histological characteristics of the main inflammatory myopathies. A and B) HE stains in a patient with dermatomyositis demonstrate perivascular infiltrates (arrows) in A and perifascicular fiber atrophy (arrows) in B. C) HE stain showing endomysial lymphocytic infiltration (arrow) in a patient with polymyositis. D) MHC-I stain in a patient with polymyositis showing a strong diffuse brownish color. E) HE stain in a patient with IBM demonstrating rimmed vacuoles (arrows). F) HE stain showing necrosis (arrows) in a patient with immune-mediated necrotizing myopathy.

The clinical scenario of weakness, typical skin lesions and elevation of CK allows a diagnosis and the beginning of treatment, before performing a muscle biopsy. In cases of diagnostic doubt, a biopsy can be performed. Muscle tissue demonstrates inflammatory infiltration, predominantly CD4 T cells, and macrophages, surrounding blood vessels (perivascular infiltrates) and invading the perimysium (perimysial infiltrates) (Figure 4A-B)^28^. However, some patients do not have infiltrates but have prominent necrosis, especially in perifascicular regions, which may be indistinguishable from immune-mediated necrotising myopathy^7^. Perifascicular atrophy and perifascicular increase of MHC class 1 labeling are characteristic but are no longer considered pathognomonic^5^. Recently, type 1 interferon (IFN1) signature and perifascicular positivity for myxovirus resistance protein A (MxA) by immunohistochemistry have been specifically associated with dermatomyositis and are potential markers on muscle histology for specific diagnosis^29^.

Neoplastic investigation is recommended, both in adults and in the juvenile form. The incidence of neoplasms in DM is increased by five to seven times when compared to the general population, with a reported frequency of 9 to 32%^30^ ^,^ ^31^. 

### Polymyositis

Polymyositis manifests in adults (rarely in children), with proximal weakness, high CK (10 to 50 times the value of reference), usually without myalgia^12^. The disease progresses over weeks to months, and therefore is different from rhabdomyolysis, which has an acute and painful onset. Polymyositis, as a distinct entity, is considered uncommon, and its existence has been questioned. Most patients previously classified in this group, following new clinicoseropathological knowledge, are categorized in another IIM^27^.

There are no skin lesions and therefore diagnosis simply with clinical criteria is not possible. Unlike DM, muscle biopsy in PM is mandatory, making a differential diagnosis with IBM, DM with atypical skin lesions, muscular dystrophies, IMNM, and rare forms of ASS. In the muscle, there is an inflammatory infiltrate between the muscle fibers and an increase in CD8-T-lymphocytes, with invasion of non-necrotic fibers (Figure 4C-D)^28^. The pathophysiology is different from DM, and it is worth emphasizing that DM is not polymyositis with skin lesions.

### Inclusion body myositis (IBM)

IBM is a late-onset form of myopathy, and though classified in the group of IIM, it has a degenerative physiopathology. It is considered the most common form of myopathy in patients over 50 years of age and is more common in men, in a 2:1 proportion^32^. Clinically, the disease affects predominantly the quadriceps and the gastrocnemius muscles in the lower limbs and the finger flexors in the upper limbs^9^. The onset is slowly progressive, and the muscle involvement is usually asymmetrical^3^. 

Magnetic resonance imaging shows signal alteration in T1-weighted sequences, mainly in the distal portion of the quadriceps, meaning fat-infiltration of this muscle, and corresponding finds may also be detected by ultrasound^33^. 

The skeletal muscle abnormalities include a T-cell-mediated endomysial inflammatory reaction in association with degenerative changes characterized by the presence of rimmed vacuoles (Figure 4E), intracytoplasmic inclusions formed by the accumulation of abnormal proteins, 𝛽-amyloid deposits, and mitochondrial changes^9^ ^,^ ^34^. 

Autoantibodies against cytosolic 5ʹ-nucleotidase 1A (NT5C1a) has been studied, but its sensitivity (around 30%) and questionable specificity (up to 20-30% of patients with other reumatological conditions may have this autoantibody) do not yet allow its recommendation in clinical practice^34^. In the absence of a serological marker, muscle biopsy is still mandatory in suspected IBM patients. On the other hand, serologies for chronic viruses, such as HIV, HTLV, and hepatitis C and B must be done, because IBM might develop from these viral infections, resulting in a typical disease course, except for a younger age of onset^34^. 

Recently, it has been shown that most IBM patients present T-cells expressing CD57, a surface marker of T-cell large granular lymphocytic leukemia, and up to one-third of IBM patients in a cohort met criteria for this type of leukemia, with cytopenias^35^. Although, there is no consensus regarding this point, we think that hematological evaluation may be warranted, and blood cell counts, as well as blood flow cytometry must be considered. 

### Immune-Mediated Necrotizing Myopathy

IMNM is a myopathy with peculiar clinicopathological features, occurring much more frequently than PM, and presenting in a large range of age^14^. Onset may be acute or subacute, with high levels of CK, which can be triggered by viral infection, cancer, and statin use^36^. However, it can develop without any of these factors present. In children, the disease may have a chronic slowly progressive course, and the patients can be initially diagnosed with muscular dystrophy^37^. Pathologically, muscle necrosis, presence of CD68+ macrophages and little or no lymphocytic inflammation are observed (Figure 4F)^36^.

The 224th ENMC International Workshop categorized IMNM into three subgroups according to autoantibodies: antisignal recognition particle (SRP) IMNM, anti3-hydroxy-3-methylglutaryl-coenzyme A reductase (HMGCR) IMNM, and seronegative IMNM^36^. This classification is important not only for diagnosis, but also for the prognosis and planning of the treatment. Anti-SRP patients usually have a more severe muscle involvement and a more resistant response to immunosuppressors. Anti-HMGCR were initially found in IMNM patients who were taking statins, but now we know there are many patients with this autoantibody without statin exposure. The association with malignancy is not consistent in anti-SRP and anti-HMGCR subgroups, but the risk is increased in seronegative patients^38^.

### Antisynthetase syndrome

ASS is classically characterized by the presence of inflammatory myopathy, interstitial lung disease and joint involvement, although other findings may be present, such as fever, "mechanic's hands", and Raynaud's phenomenon^39^ ^,^ ^40^. Serologically, it is characterized by the presence of antibodies against aminoacyl-transfer RNA (tRNA) synthetases. The list of antisynthetase autoantibodies has been evolved over the last decades and three are the most studied and recognized ones: anti-Jo-1 (antihistidyl), anti-PL-7 (antithreonyl), and anti-PL-12 (antialanyl) (Table 1). Now the presence of one of the ASS-specific autoantibodies is considered a necessary condition to classify the patient as having ASS^5^ ^,^ ^16^ ^,^ ^41^. 

Some authors consider ASS as being in a broader group, known as overlap myositis. In fact, patients with ASS often have signs and symptoms that overlap with other connective tissue disorders (CTD), such as Raynaud’s phenomenon, interstitial lung disease, arthritis, gastroesophageal reflux disease, and general systemic symptoms^17^. Additionally, patients with ASS may present skin rashes and, histologically, the inflammatory involvement resembles that of dermatomyositis. A muscle biopsy demonstrates T-cell and macrophage infiltrations and perifascicular atrophy or perifascicular necrosis^7^. Without serological evaluation, ASS patients may be misdiagnosed either as PM, IMNM or, if there is skin lesion or perifascicular atrophy on histology, as DM.

Importantly, not all patients with ASS have muscle weakness. For instance, muscle involvement is more common in anti-Jo-1 patients, whereas pulmonary involvement is more common in the presence of anti-PL-12 and anti-PL-7, with some patients presenting no clinical weakness^5^. Morbidity and mortality are associated with rapidly progressive pulmonary involvement^39^ ^,^ ^41^. 

### Overlap myositis

Overlap myositis is the association of inflammatory myopathy with at least one clinical overlap feature of a connective tissue disorder, such as systemic lupus erythematous (SLE), systemic sclerosis (SS), rheumatoid arthritis, or Sjögren’s syndrome^14^. In these cases, myositis appears as an additional manifestation of a more complex rheumatological disease. As mentioned above, ASS may be considered as an overlap myositis, because of intersect characteristics of myositis, skin lesions, lung involvement, and Raynaud’s phenomenon^17^.

There are also some autoantibodies associated to this kind of presentation. The most common of these antibodies are anti-PM (polymyositis) /Scl (scleroderma) and anti-U1-RNP (ribonucleoprotein) antibodies. These are observed in up to 20% of all patients with myositis^4^. Anti-PM/Scl is associated with upper limb weakness predominance and anti-U1-RNP is associated with interstitial lung disease^5^. Muscle biopsy may present perivascular inflammation, perifascicular necrosis and MHC-I increase. 

Interestingly, if we consider ASS as part of a wider overlap myositis group, this will represent approximately 50% of all patients with myositis, followed by dermatomyositis and IMNM, whereas polymyositis is the rarest form of IIM^27^. 

## DIFFERENTIAL DIAGNOSIS WITH MUSCULAR DYSTROPHY

Some clinical situations can cause mistakes in the differentiation between IIM and muscular dystrophy. There are several forms of muscular dystrophy that may present an inflammatory infiltrate, such as fascioscapulohumeral dystrophy or girdle dystrophy due to dysferlin or anoctamin deficiency^42^ ^,^ ^43^. In these cases, the onset can mimic an acquired myopathy, with proximal weakness of subacute installation and very high CK. In cohorts of patients with dysferlinopathies, up to 25% of the patients are initially defined as having inflammatory myopathy^44^. To differentiate these conditions, in addition to reassessing the clinical phenotype and the response or not to immunosuppressants, some biopsy findings may help. The CD8 lymphocyte infiltrates and diffuse increase in MHC class I labeling are more frequent in inflammatory myopathies, while in dystrophies, lymphocytic infiltrates are focal, and rarely have increased labeling for CD8 or MHC class I. Furthermore, in dystrophies, the finding of fibrosis between the fibers is more frequent and immunohistochemistry may be diagnostic by revealing the absence of muscle proteins (such as dysferlin, sarcoglycans, dystrophin). 

On the other hand, IMNM caused by anti-SRP and anti-HMGCR antibodies may present with an insidious course, clinically confusing with muscular dystrophy, even with muscle biopsy showing some degree of connective tissue increase, and with little response to corticosteroids^42^. In these cases, the presence of serum autoantibodies and the finding of necrosis as the main histological characteristic, associated with an increase in immunohistochemical staining for macrophages (CD68) and MHC class I, are the elements that favor the diagnosis of IMNM.

## HOW TO PLAN THE TREATMENT

**Figure 5.**
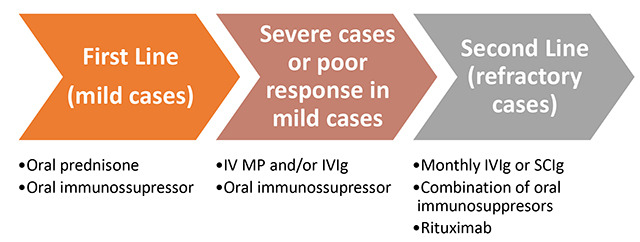
Schematic flowchart for IIM treatment.

IV: intravenous; MP: Methylprednisolone; IVIg: intravenous human immunoglobulin; SCIg: subcutaneous immunoglobulin.

Due to the rarity and heterogeneity of IIM, evidence guiding how to plan patients’ treatment is limited, and essentially based on case reports, series, expert opinions, and a few non-high-quality randomized trials (Figure 5)^45^. Treatment in clinical practice consists of corticosteroid therapy, and prednisone 0.5 to 1 mg/kg is usually prescribed in mild cases, over a period of four to eight weeks with serum CK levels monitoring and muscle strength evaluation, before tapering or adding another immunosuppressor^7^ ^,^ ^46^. Intravenous pulses of methylprednisolone (MP) 1,000 mg daily for three to five days is recommended for more severe cases associated with functional disability, dysphagia, interstitial lung disease, ulcerated skin lesions or a rapidly progressive course^46^. In even more dramatic cases with significant weakness (for example, patients unable to walk unassisted), respiratory involvement, severe dysphagia or refractory to initial corticosteroid treatment, intravenous human immunoglobulin (IVIg), at a dose of 2 g/kg, divided over two to five days may be used^46^. Both pulse therapies (MP and IVIg) may be repeated monthly until there is a good clinical response, and in some case, to maintain remission^3^.The total duration of treatment is not consensual, but most experts start gradual reduction of corticosteroids after disease remission, followed by progressive reduction of other immunosupressors, in a period over one to two years following disease control^46^. 

Immunosuppressants are indicated in practically all cases for glucocorticoid sparing and to avoid relapses during corticosteroid tapering^3^ ^,^ ^47^. The most used drugs are azathioprine (2 to 3 mg/kg/day), methotrexate (10 to 25mg weekly), mycophenolate mofetil (2 to 3 g/day) and cyclosporine (3 to 5 mg/kg/day)^7^ ^,^ ^46^. The choice is based mainly upon the experience of the prescriber and adverse effects profile of each patient. 

Immunobiologic agents that have been approved for the treatment of other immune diseases may be considered in refractory cases. The most used is the rituximab (anti-CD20 antibody) at a dose of 2g^46^. It seems effective in some patients with dermatomyositis, polymyositis, or necrotizing autoimmune myositis^3^. A multicenter placebo-controlled study involving 200 patients did not demonstrate a response in the eighth week (the primary endpoint), but at week 44, when all had received rituximab, 83% presented the definition of improvement in treatment^48^. Another option proposed for refractory patients is to maintain chronically subcutaneous immunoglobulin^49^.

For IBM patients there is no defined treatment. Available studies have failed to demonstrate a real benefit in using immunosuppressants^34^. Currently, intravenous human immunoglobulin may be used for the treatment of dysphagia^3^.

Physical exercise programs under supervision are safe in all types of IIM^50^. The patient must be encouraged to start a routine of exercises to increase strength, reduce disability, so helping a faster improvement^51^.

In conclusion, advances have been seen in IIM over the last decade. There are new concepts for classification, especially based on the development in serodiagnosis, with many autoantibodies currently known. Probably, two-thirds of IIM patients have an autoantibody, which may make the subgroups more homogenous, improving not only the diagnosis, but prognosis and treatment planning. Knowledge in this field is continuing and rapidly evolving, so neurologists must be updated to better assist their patients with these treatable myopathies.
